# Cell death induced by ozone and various non-thermal plasmas: therapeutic perspectives and limitations

**DOI:** 10.1038/srep07129

**Published:** 2014-11-20

**Authors:** Oleg Lunov, Vitalii Zablotskii, Olexander Churpita, Eliška Chánová, Eva Syková, Alexandr Dejneka, Šárka Kubinová

**Affiliations:** 1 Institute of Physics AS CR, Prague, Czech Republic; 2 Institute of Macromolecular Chemistry AS CR, Prague, Czech Republic; 3 Institute of Experimental Medicine AS CR, Prague, Czech Republic

## Abstract

Non-thermal plasma has been recognized as a promising tool across a vast variety of biomedical applications, with the potential to create novel therapeutic methods. However, the understanding of the molecular mechanisms behind non-thermal plasma cellular effects remains a significant challenge. In this study, we show how two types of different non-thermal plasmas induce cell death in mammalian cell cultures via the formation of multiple intracellular reactive oxygen/nitrogen species. Our results showed a discrepancy in the superoxide accumulation and lysosomal activity in response to air and helium plasma, suggesting that triggered signalling cascades might be grossly different between different plasmas. In addition, the effects of ozone, a considerable component of non-thermal plasma, have been simultaneously evaluated and have revealed much faster and higher cytotoxic effects. Our findings offer novel insight into plasma-induced cellular responses, and provide a basis for better controlled biomedical applications.

Plasma is determined as a partially or completely ionized medium, representing a coalition of various chemical entities, ultraviolet radiation and heat^1^. Non-equilibrium plasma is generally categorized according to its gas temperature as thermal or non-thermal. Currently, interest regarding the application of non-thermal plasmas in biomedical research is rapidly growing^2^,^3^. Due to the increasingly difficult problem of emerging antibiotic resistant microorganisms^4^,^5^, there is an indispensable need for the design and development of novel alternatives to antibiotics. In this regard, non-thermal plasma represents an important and effective alternative approach in overcoming resistant microorganisms^6^,^7^.

Tremendous progress in understanding the physical plasma phenomena has resulted in the development of new non-thermal plasma sources. Of late, it has been possible to produce non-thermal plasma utilizing several methods, including dielectric barrier discharge (DBD), atmospheric pressure plasma jet, plasma needle, and plasma pencil^8^. Furthermore, it is relatively easy to manipulate plasma density, temperature, and composition to control plasma products. This allows the chemical composition of the plasma to change with ease upon the application of different gases, such as helium, argon, nitrogen, heliox (a mix of helium and oxygen), and air. The resulting composition of plasma is very complex consisting of charged particles (electrons, ions), electronically excited atoms and molecules (including ozone – O_3_), radicals, and UV photons^8^,^9^.

Recently, a burst of studies have shown the potential use of non-thermal plasma in a wide variety of biological and medical applications, such as wound healing^10^,^11^, microorganisms deactivation^2^, blood coagulation^3^, dental cavity treatment^12^, angiogenesis suppression^13^, and cancer treatment^13^. Furthermore, such studies have sometimes ascribed a bewildering variety of biological effects to non-thermal plasma. Moreover, the generation of devices required to produce non-thermal plasma and apply it to tissue are relatively simple and flexible. Therefore, possible applications of non-thermal plasma as a novel postulated therapy has attracted attention^3^.

Although the biological effects of non-thermal plasma have been investigated thoroughly in recent years, its cellular targets as well as the molecular or biophysical foundations of its alleged biological effects remain generally unknown. Several lines of research have shown the potential role of reactive oxygen (ROS) and nitrogen species (RNS) in the underlying biological effects of plasma^14^,^15^,^16^. The importance of ROS involved in cell signalling has been implicated in many biological systems from bacteria to mammalian cells^17^. ROS recruits many different mitogen-activated protein kinases (MAPK) resulting in the activation of cell signalling cascades^18^. Taking into account ROS generation using non-thermal plasma, there is no surprise that plasma-induced biological effects span from increased proliferation^14^,^19^ to cell death by necrosis^20^ or apoptosis^15^,^16^ through to the formation of intracellular ROS^14^,^15^.

Most biomedical plasma applications require well defined and controlled interactions between non-thermal plasma and living cells. However, our understanding of the interactions between plasma and living cells, as well as the underlying mechanisms remains rather limited. As of yet, no comparative study has been thoroughly performed that observes the effects of different chemical compositions of plasma on the physiological functions of the cells. Moreover, non-thermal plasma is capable of producing significant amounts of ozone^8^,^9^, which is known to have very aggressive effects on cells^21^,^22^. However, the information concerning comparison of the biological effects of non-thermal plasma and ozone are basically lacking. Indeed, ozone can directly induce cell death via the accumulation of intracellular oxidants. It has been shown that oxidative stress induced by ozone may initiate redox-sensitive MAPK signalling resulting in different biological processes, including inflammation and cell death^21^,^23^. Interestingly, ozone produced by non-thermal plasma may remain for a long time even after turning off the plasma generator^24^. Hence, it is of great importance to compare and discriminate cellular effects triggered by plasma from those induced by ozone.

Therefore, the aim of this study is to investigate and compare the effects of two different non-thermal plasmas and ozone on physiological and pathophysiological cellular functions, as well as gaining information on the potential molecular targets of non-thermal plasma.

## Results

### Characterization of the plasma jet

**Figure 1:**
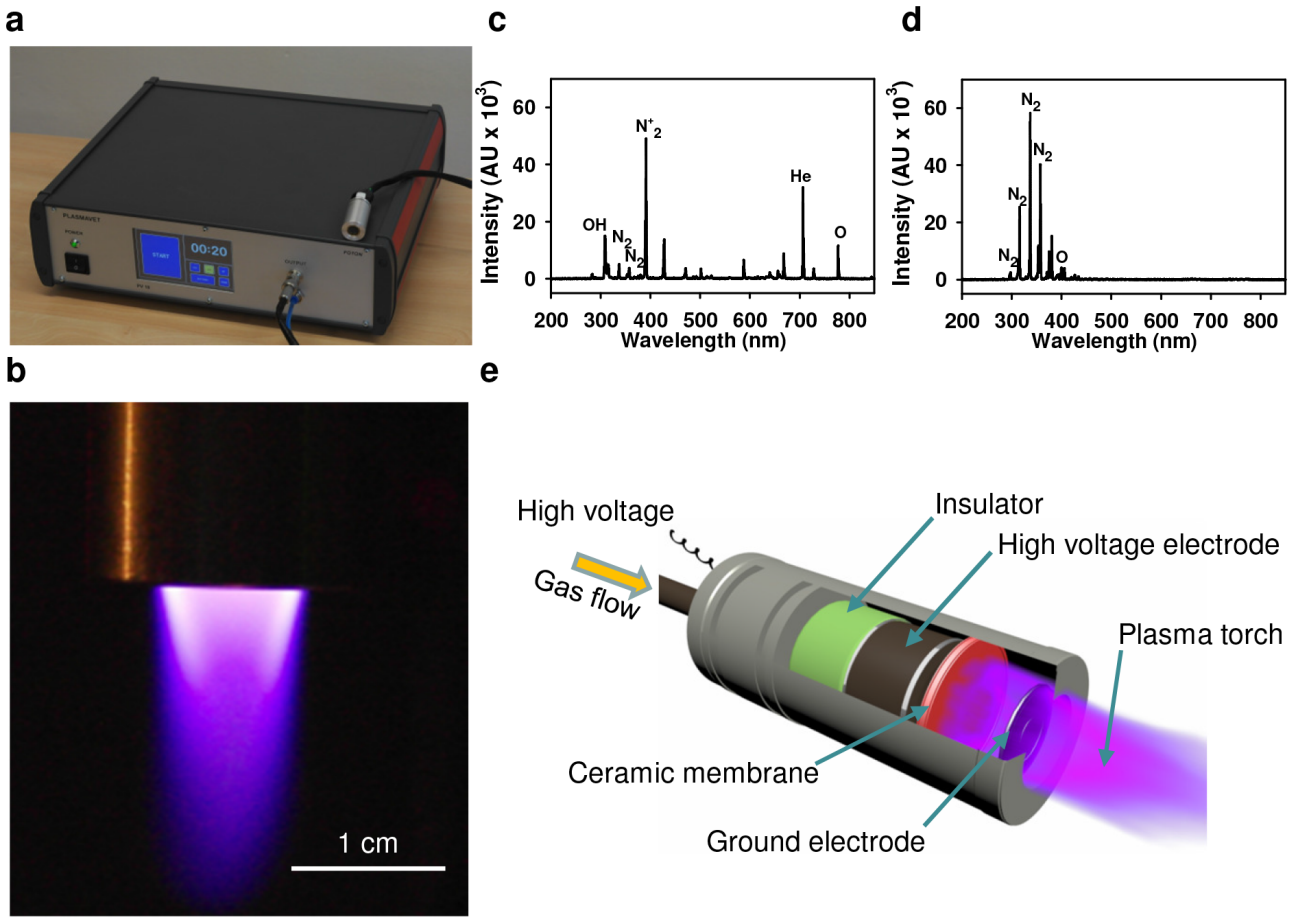
Scheme and physicochemical characterization of the plasma device. (a) Photograph of the fabricated plasma jet system. (b) An image of the plasma torch. Optical emission spectrum of helium (c) and air (d) plasma during discharge. (e) Schematic diagram of plasma nozzle.

We utilized a single jet non-thermal plasma system with 200 kHz and 600 V of applied AC power to study the effects of plasma treatment on mammalian cells^25^. A characteristic picture of the plasma system with an image of a plasma torch is shown on Figure 1a, b. Non-thermal plasma was generated in a specifically designed nozzle bearing a plasma generation module (Fig. 1e). The designed system enables the chemical composition of the plasma to easily change with the application of distinct gases (air, helium, nitrogen, etc.). Generally, the plasma jet system operated under a specific atmospheric pressure is able to generate a variety of chemically active species, particularly oxygen and nitrogen atoms^8^,^9^,^26^. Different types of plasma devices (dielectric barrier discharge (DBD) or direct plasma generators) are also known to be used in biomedical applications. As a rule, in such devices the plasma treated sample is connected to one of the electrodes and as a result has a non-zero potential. Beside this, a high voltage (a few kV) is usually applied to electrodes in such devices. Both of these factors are unfavourable for the study of interactions between plasma ions and cells because the electric field itself has a direct impact on cell functionality, e.g. cell membrane electroporation effect. In spite of this, DBD and direct non-thermal plasma sources are also used for a variety of biomedical applications^7^,^9^,^27^. However, to separate the direct effects of the electric field and plasma – for cell interaction it is better to use generators in which plasma is produced relatively far from the treated cells. To study differentiated and comparable cellular effects, we exploited two types of different plasmas, i.e. air and helium plasma. First of all, we used optical emission spectrum analysis to validate the plasma composition in terms of particles and radicals generated by the air and helium plasma systems (Fig. 1c, d), which can mediate the cellular effects of plasma. Optical emission spectroscopy, which was performed over a wide range of wavelengths from 200 nm to 900 nm, revealed distinct chemical differences between the two types of plasma (Fig. 1c, d). In an effort to monitor the reactive species environment produced by the jet array in the vicinity of the cell cultures, optical emission spectra were recorded with the spectrometer OceanOptics USB 4000-UV-VIS. The spectra were recorded in the longitudinal geometry with the device probe in an axial position at a distance of 10 mm from the nozzle outlet. Acquisition time was 1 sec. Helium and air plasmas generated by microdischarges in porous ceramics were investigated. Molecular OH, atomic (He, O) radicals, and other active species, e.g. N_2_, were identified (see Fig. 1c). Helium plasma of a similar spectra was successfully applied for wound healing as reported in^28^. The spectrum of air plasma is similar to that described in^27^. The emission spectrum of the air plasma is shown in Fig. 1d. The peaks correspond to nitrogen molecules. A small peak at 400 nm corresponds to atomic oxygen. The total Helium or air flow through the micronozzle was set to 4 L min^−1^ for each gas. The emitting plasmas were spatially localized well, showing the applicability of the proposed plasma reactor for controlled treatments of cells and tissue. The gas temperature of such plasma jets was measured by a K type thermocouple at a 10 mm distance from the nozzle and the temperature did not exceeded 36°C over the entire treatment time. Thus, a limited gas temperature implies the absence of thermal damage on living cells or tissue during plasma treatments. These results imply that both plasmas may represent an important source of reactive oxygen and nitrogen species production.

### Antimicrobial effects of plasma

**Figure 2:**
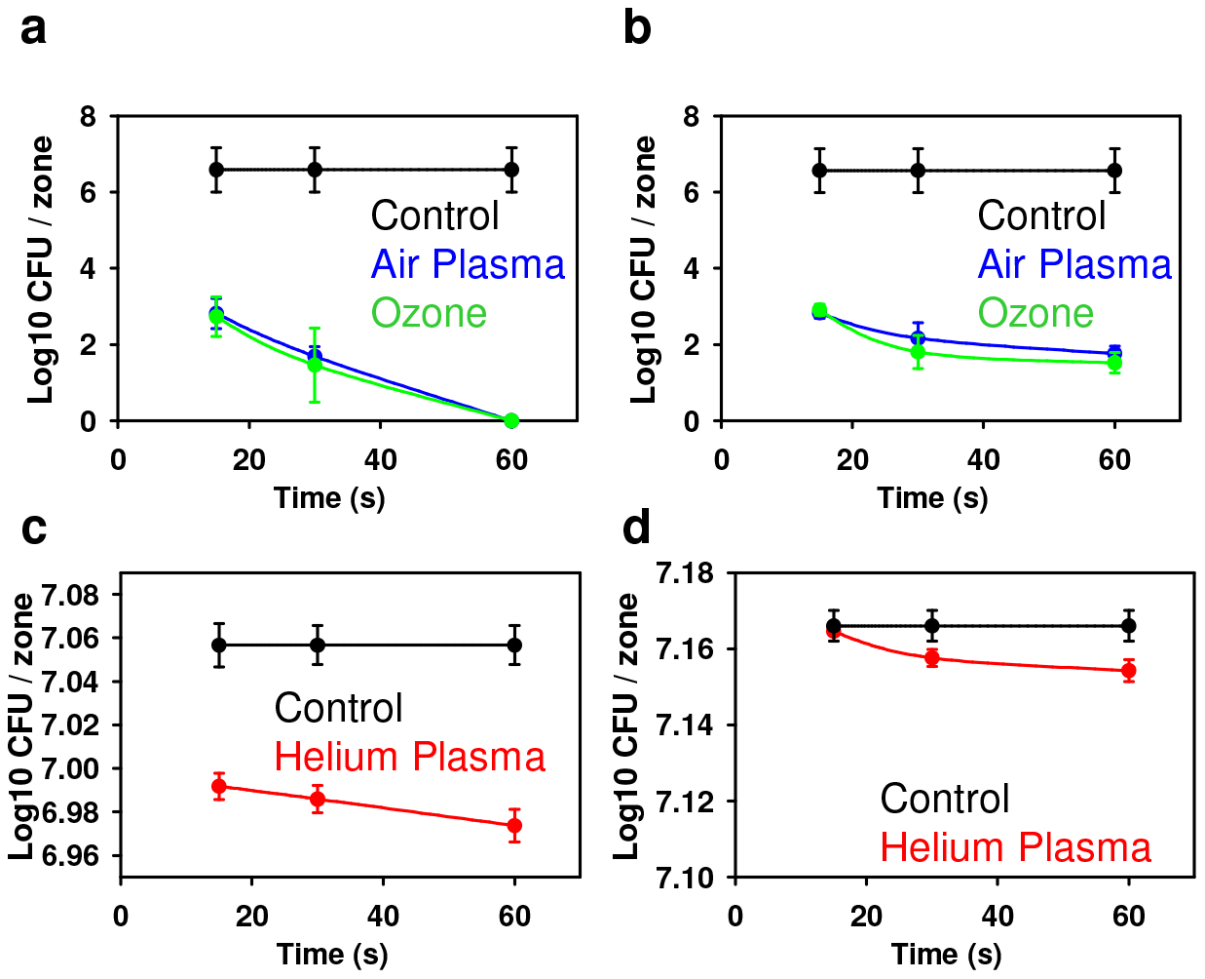
Inactivation of different bacterial strains *P. aeruginosa* and *S. aureus* by the application of separate air and helium plasma or ozone. The inactivation (number of colony forming units) of (a) *P. aeruginosa* and (b) *S. aureus* after treatment for indicated periods of time with air plasma or ozone. The inactivation (number of colony forming units) of (c) *P. aeruginosa* (d) *S. aureus* after treatment for indicated periods of time with helium plasma. Data are presented as mean ± SD, n = 4.

Non-thermal plasmas have been successfully shown to inactivate microorganisms^29^. The presence of a variety of microbicidal active agents in plasma makes it an appropriate tool for microbial decontamination^30^. In the light of a growing antibiotic resistance microbial problem, plasma treatment may be a promising technique to overcome this challenge. Therefore, we tested antimicrobial effects of air and helium plasmas in comparison with ozone on two types of bacteria, *S. aureus* and *P. aeruginosa*; these bacterial strains are often used as models of antibiotic resistant Gram-positive and Gram-negative bacteria. As one can see from Figure 2, for all treatment variations we found statistically significant antibacterial effects compared to the untreated control. Indeed, the bactericidal efficacy depended on treatment time and differed between air and helium plasma. Interestingly, bacterial inactivation of both strains *P. aeruginosa* and *S. aureus* by air plasma and ozone was the same (Fig. 2a, b), having nearly 99.99% antimicrobial efficacy. However, dramatically lower antimicrobial efficacy was found for the helium plasma (Fig. 2c, d). Both ozone and air plasma had comparable bacterial inactivation effects on both Gram-positive and Gram-negative bacteria (Fig. 2a, b). In contrast, helium plasma demonstrated higher efficacy in killing Gram-negative rather than Gram-positive bacteria (Fig. 2c, d).

### Analysis of cytotoxic effects induced by different plasmas and ozone

**Figure 3:**
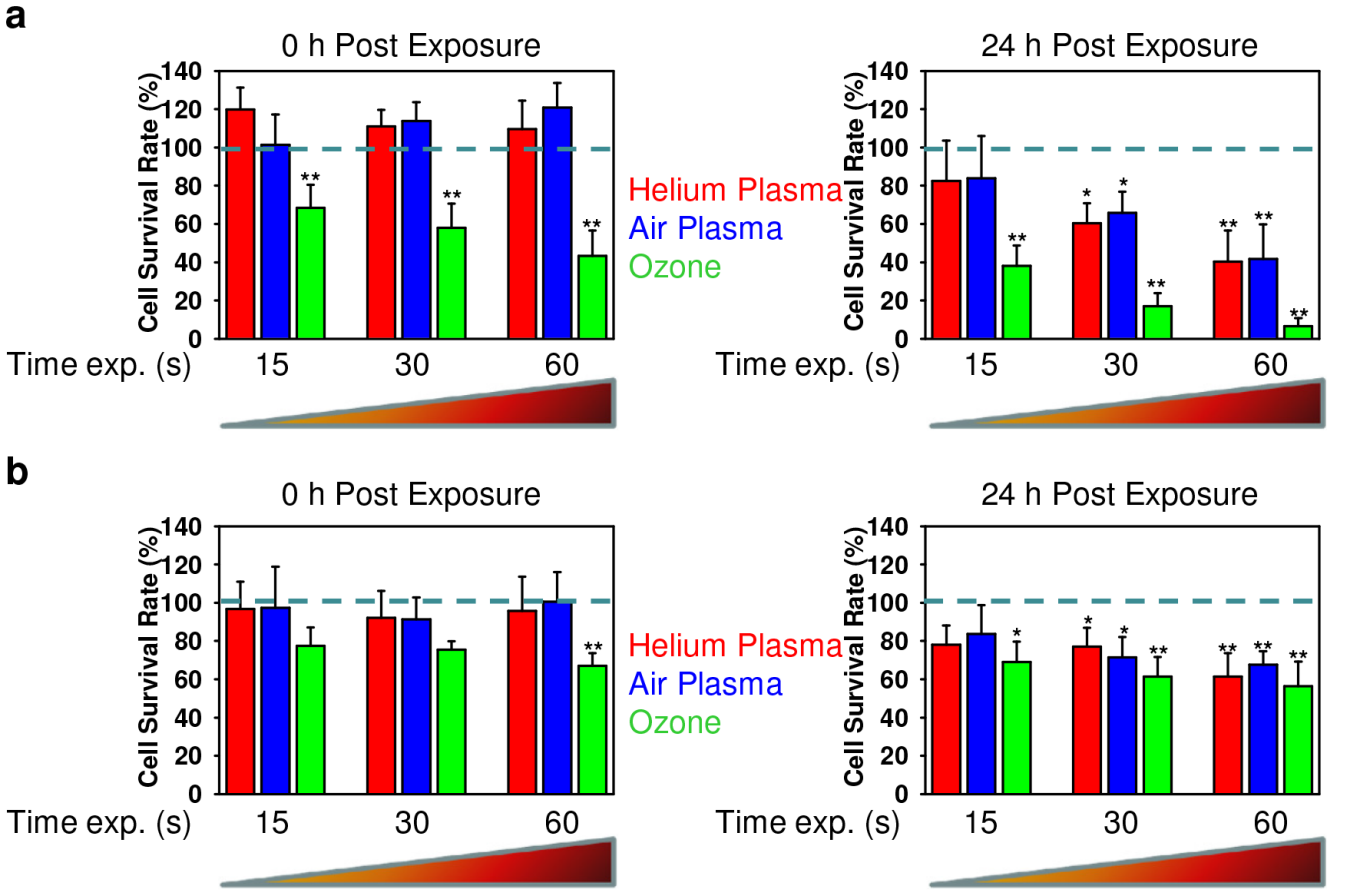
Time-dependent effects of helium, air non-thermal plasmas and ozone on 3T3 and glioma cells. (a) Cell viability as detected by the WST-1 assay of 3T3 fibroblasts treated with air, helium plasma or ozone for indicated time periods, measured 0 or 24 h after exposure. The data were normalized to control values (no particle exposure), which were set as 100% cell viability. (b) Cell viability as detected by the WST-1 assay of C6 glioma cell line treated with air, helium plasma or ozone for indicated time periods. Analysis was carried out as described in (a). *P <0.05 **P <0.01, mean ± SD, n = 3.

There are several studies that demonstrate the acute cytotoxicity of two distinct types of plasmas on living cells^14^,^16^,^24^,^31^,^32^. However, a comparison of cytotoxic effects of different plasmas on the same cellular model system has not been performed. Furthermore, non-thermal plasma has been shown to produce considerable amounts of ozone^8^,^9^,^14^. It is well known that ozone is an extremely reactive gas and exposure to it results in acute cytotoxic effects^21^,^22^,^33^. For superficial cancer treatment and wound healing it is important to know the time and dose dependent effects of plasmas on pathogenic (cancerous) cells and healthy tissue. Here we analyse the time-dependent effects of the air and helium plasmas as well as ozone on glioma cells and 3T3 fibroblasts. It is worth noting that two lines of research currently intensively investigated are cancer treatment and wound healing by non-thermal plasma^3^. Indeed, cell lines of different origin and varying degrees of differentiation have frequently been used to model wound healing and cancer condition since primary tissue cells cannot be readily expanded *ex vivo*. Further, 3T3 fibroblast cell cultures are commonly used as a model of wound healing^34^. Due to their advanced differentiation state, the C6 glioma cell line is often used as a surrogate of *in vitro* glioblastoma tissue^35^. Therefore, we utilized these two commonly accepted cell cultures as model systems for our experiments using non-thermal plasma. The comparison of cellular effects of ozone and non-thermal plasma is of particular interest practically. This prompted us to perform a comparative study of the cellular effects triggered by the two types of plasma and ozone. As expected the effects of plasma on the viability of both glioma and 3T3 cells were dose- and time- dependent (Fig. 3). There was no significant difference between air and helium plasma in terms of triggering cytotoxicity (Fig. 3). However, ozone appeared to induce higher instances of cell death (Fig. 3a). Even short term exposure triggered cell death, whereas air and helium plasma were unable to induce any signs of cytotoxicity (Fig. 3a). Both air and helium plasmas showed time-delayed cytotoxic effects only 24 h post exposure (Fig. 3a). Interestingly, the effects of both plasmas and ozone were cell type specific. Indeed, the glioma cell line (Fig. 3b) was much less affected by ozone or either plasmas in comparison with fibroblasts (Fig. 3a). However, for sufficient killing of glioma cells it may be possible that a higher dose or longer exposure is needed, as far as it has been shown that the peak of glioma cell death occurs after 72 h post plasma exposure^36^. We found that ozone possesses a high level of toxicity and fasted cellular responses within 24 h post plasma exposure, furthermore we focused our studies on this time scale to better reveal differences between plasmas and ozone treatment.

**Figure 4:**
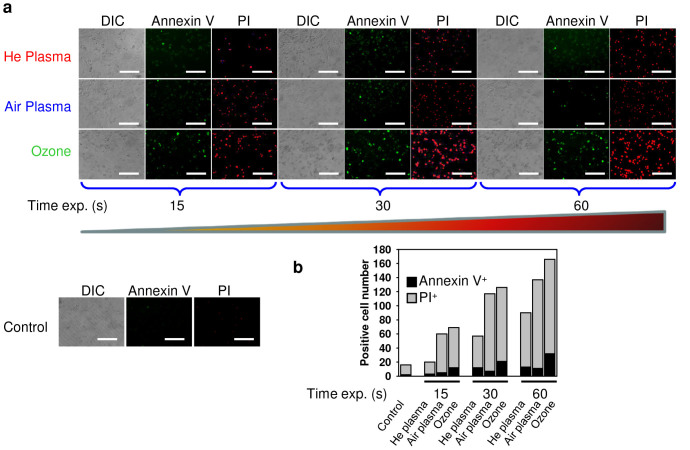
Analysis of apoptotic cell death. (a) 3T3 fibroblasts were treated with air, helium or ozone, then 4 h after treatment cells were labelled with annexin V – green dye and propidium iodide – red dye. Labelled cells were imaged with fluorescence microscopy. (b) Microscopy quantification of annexin V and propidium iodide-positive cells. Scale bar 200 µm.

Exposure of fibroblasts to air, helium plasmas or ozone induced only very low signs of early apoptosis, namely translocation of phosphatidylserine to the outer cell membrane leaflet, as measured by binding of FITC-labeled annexin V (Fig. 4a, b). Instead, a concomitant increase in membrane permeability, as shown by propidium iodide exclusion (Fig. 4a, b), was the predominant effect induced by both plasmas and ozone. Indeed, the permeability of the cell membrane affects dramatically on the ability of propidium iodide to enter a cell^37^. Having an intact plasma membrane living or early apoptotic cells are not permeable for propidium iodide. In contrast, late apoptotic or necrotic cells have plasma and nuclear membranes with decreased integrity, allowing propidium iodide to pass through the membranes^37^. Thus, our observations of predominant propidium iodide nuclear staining suggest either late stage apoptotic or necrotic cell death induced by both plasmas and ozone. Consistent with cytotoxicity data, ozone was more reactive in triggering propidium iodide incorporation into the cell nucleus in comparison with air and helium plasma (Fig. 4). It is worth noting that helium plasma produced less propidium iodide positive cells in comparison with air plasma (Fig. 4).

### Changes in cell membrane integrity upon plasma treatment

**Figure 5:**
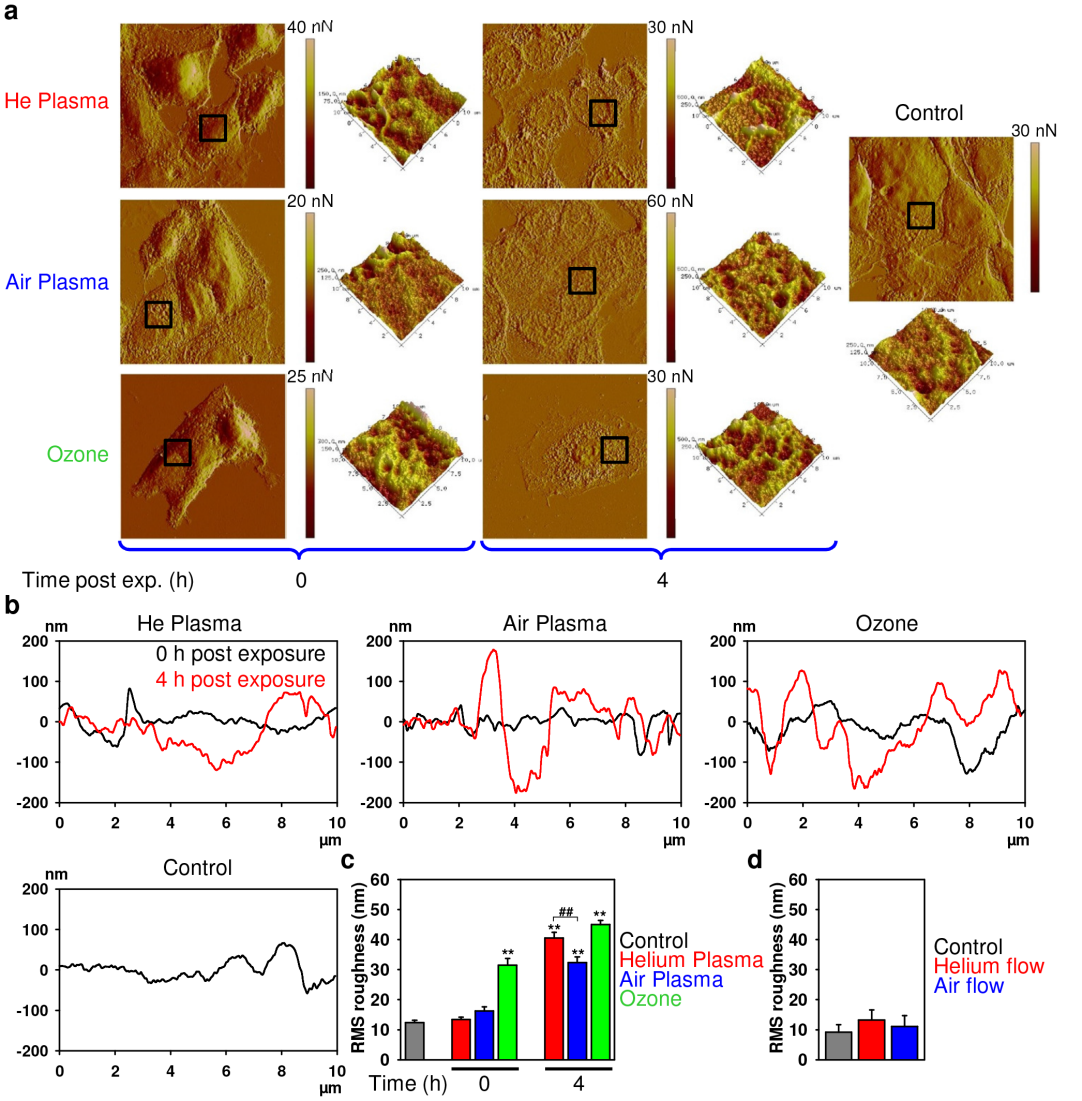
AFM analysis of cell membrane integrity. (a) 3T3 fibroblasts were treated with air, helium or ozone for 30 s, and then 0 or 4 h after treatment cells were fixed and imaged with AFM microscopy. Images represent a 90 × 90 µm area of scan. The black square areas indicate where a 3D 10 × 10 µm scan has been performed. (b) Representative surface profiles of 10 × 10 µm scanned area of the cell treated as in (a). (c) AFM microscopy quantification of membrane roughness of the cell treated as in (a). (d) AFM microscopy quantification of membrane roughness of 3T3 fibroblasts were treated with air, helium flow (no plasma treatment), and then 4 h after treatment cells were fixed and imaged with AFM microscopy.

It is well known that propidium iodide does not penetrate through the plasma membrane of viable or early stage apoptotic cells^38^; only cells that have lost plasma membrane integrity are permeable to this dye. Therefore, we investigated in detail membrane damage induced by both plasmas and ozone utilizing atomic force microscopy (AFM), for details see section “Methods”. In fact, short exposure of cells to air or helium plasma had no significant effect on the cell surface morphology compared to the untreated control cells (Fig. 5a). However, 4 h post exposure we were able to detect changes in membrane morphology in response to both plasma treatments (Fig. 5a). Conversely, ozone treatment induced immediate changes in the cell surface morphology, which remained unchanged for 4 h post exposure (Fig. 5a). Interestingly, the roughness of the outer cellular membrane surface increased following treatment of cells with both plasma types 4 h post exposure (Fig. 5b, c), indicating a loss of membrane integrity. Moreover, 4 h after treatment helium plasma induced significantly more profound effects on membrane roughness than air plasma (Fig. 5c). Furthermore consistent with previous data, ozone induced a greater increase in membrane roughness (Fig. 5b, c). Flow of gas alone, either helium or air, through the plasma device did not induce changes in membrane integrity or roughness (see Fig. 5d and Fig. S1 in the Supplementary Information).

### Generation of intracellular ROS and RNS by different plasmas and ozone

Previous studies have reported that non-thermal plasma is able to catalyze the generation of reactive oxygen (ROS) and nitrogen species (RNS) in living cells^14^,^15^. Indeed, high levels of ROS may induce damage to cellular structures and may finally lead to cell death^39^. Moreover, it has been analyzed that non-thermal plasma is potent in the production of extracellular ROS and RNS and mediates their delivery into the liquid phase of the cell culture medium, and subsequently their diffusion into cells^16^. Therefore, we were interested in the generation of intracellular ROS following plasma treatment.

**Figure 6:**
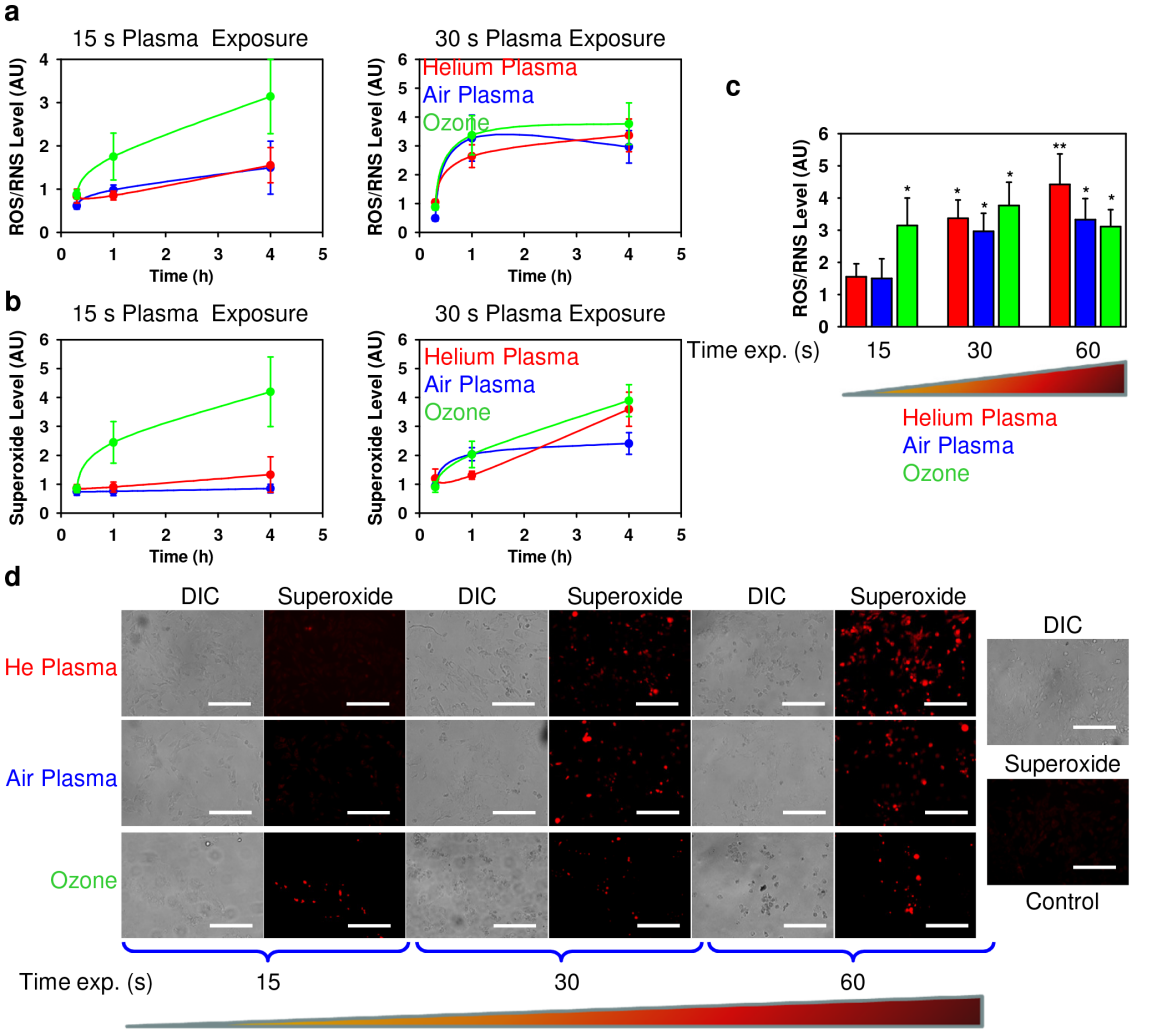
Air, helium plasma and ozone induce ROS production in 3T3 fibroblasts. Cells were exposed to air, helium plasma or ozone, followed by ROS measuring using the cellular ROS/Superoxide detection kit (Abcam). (a) Time-dependent ROS/RNS induction by air, helium and ozone. (b) Time-dependent superoxide induction by air, helium and ozone. The data are presented as mean percentage of ROS/Superoxide positive cells mean ± SEM, n = 4. (c) Assessment of total intracellular ROS/RNS levels 4 h after air, helium plasma and ozone treatment. The data are presented as mean percentage of ROS positive cells mean ± SEM, n = 4, *P <0.05 **P <0.01. (d) Air, helium plasma and ozone induce superoxide accumulation 4 h after treatment. Labelled cells were imaged with fluorescence microscopy. Scale bar 200 µm.

To address this question, we evaluated ROS/RNS generation by air plasma, helium plasma and ozone using the ROS-sensitive fluorescent assay (cellular ROS/superoxide detection assay kit). We used two distinct fluorescent probes. One probe used was indicative of cellular production of different ROS/RNS types, the other was superoxide (O_2_^−^) specific. This allowed us to monitor changes in the total ROS/RNS level as well as specifically verify the level of superoxide. As shown in Figure 6 both plasmas and ozone induced a time-dependent ROS production in fibroblasts. In line with the data on cell death assessment and cell surface morphology, ozone induced a significantly higher production of total ROS/RNS than air and helium plasmas even after short time exposure (Fig. 6a, c). Moreover, both plasmas and ozone triggered a time-dependent accumulation of superoxide (Fig. 6b). Again ozone was much more reactive than air or helium plasma following an exposure time of 15 s (Fig. 6b). However, 4 h post exposure helium plasma triggered a higher accumulation of superoxide than ozone and air plasma after 60 s of treatment (Fig. 6d), as revealed by fluorescent microscopy.

### Effects of plasma and ozone on lysosomal integrity and mitochondrial membrane potential

**Figure 7:**
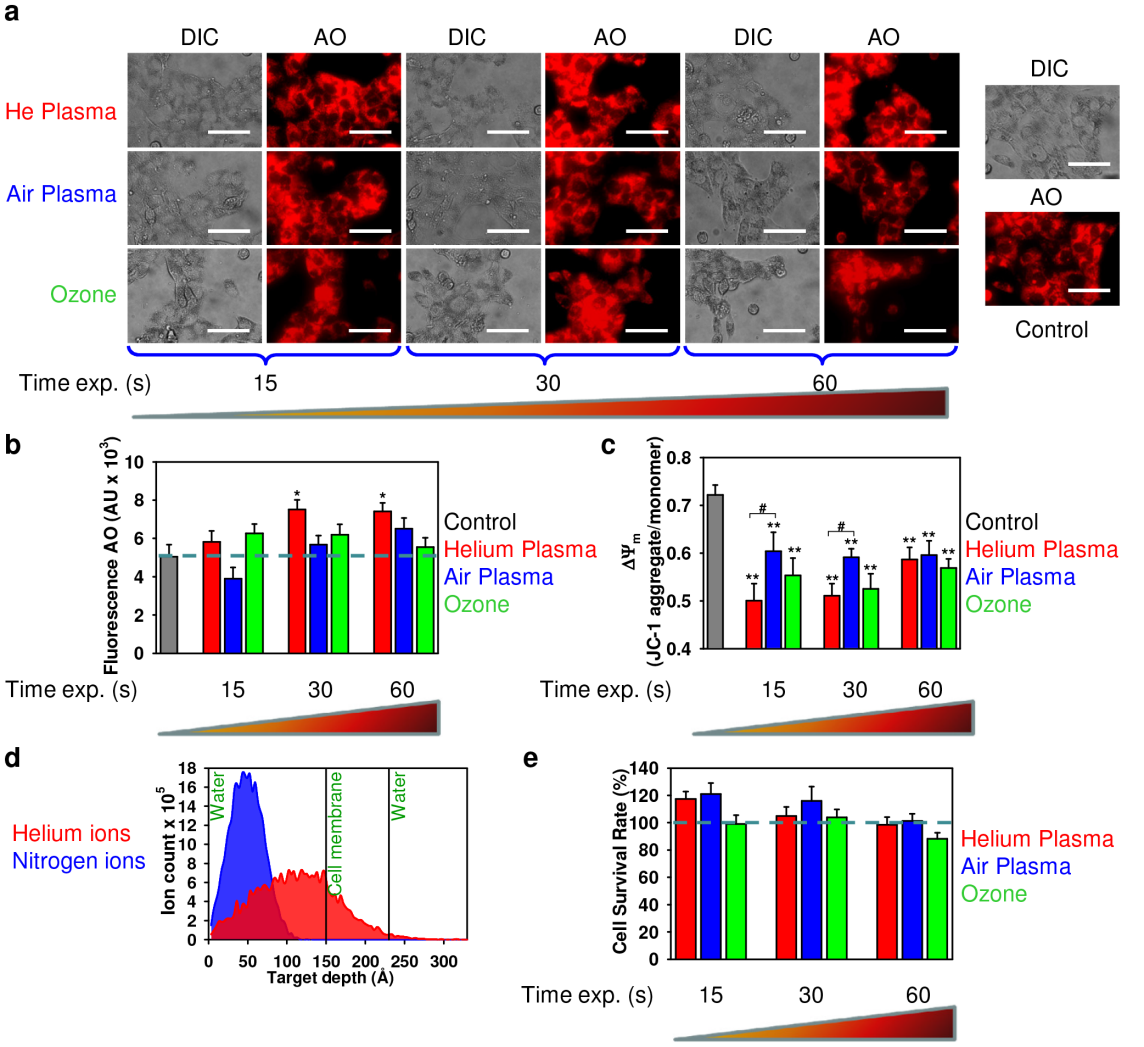
Air, helium plasma and ozone effects on mitochondrial metabolic activity and lysosomal integrity of 3T3 fibroblasts 4 h after treatment. (a, b) Assessment of plasma effects on lysosomal integrity. After treatment with plasma, cells were stained with AO. AO uptake in acidic lysosomes leads to red fluorescence, which dissipates when the dye leaves this compartment. The accompanying decrease in fluorescence intensity was analyzed with fluorescence microscopy (a) or by spectrofluorometry (b). The results are presented as the mean ± SEM of four independent experiments *P <0.05, **P <0.01 versus controls. Scale bar 100 µm. (c) Mitochondrial membrane potential (ΔΨ_m_) state in treated cells with air and helium plasma in comparison with ozone. Cells were stained with JC-1 probe, the JC-1 ratio was measured by spectrofluorometry. The results are presented as the mean ± SEM of three independent experiments, *P <0.05, **P <0.01 versus controls. (d) Results of SRIM simulations: In-depth profiles of He (red panel) and N (blue panel) ions in the three-layer H_2_O (10 nm)/membrane (8 nm)/H_2_O (10 nm). The total number of ions is 10000. (e) ROS scavenger prevents the cytotoxicity induced by air, helium plasma or ozone. Treatment with ROS scavenging agent 5 mM *N-acetyl-L-cysteine* (NAC) completely abolished the cytotoxicity of air, helium plasma and ozone 24 h after exposure. Cell viability as detected by the WST-1 assay of 3T3 fibroblasts exposed to air, helium plasma or ozone for indicated periods of time. The data were normalized to control values (no plasma or ozone exposure), which were set as 100% cell viability. *P<0.05, **P<0.01 versus controls, mean ± SEM, n = 4.

Mitochondria have been identified as a major source of cellular ROS generation^40^. Moreover, the superoxide anion is generated as a by-product of mitochondrial oxidative phosphorylation^40^. The generation and accumulation of superoxide is primarily associated with cellular toxicity and mitochondrial dysfunction^40^. It has been shown that lysosomal rupture and the associated release of lysosomal enzymes promote the mitochondrial production of oxidants^41^,^42^. Therefore, we investigated the putative role of air, helium plasmas and ozone on lysosomal destabilization. To address the possible lysosomal leakage induced by plasmas and ozone, we used the lysosomotropic dye acridine orange (AO). The accumulation of AO in a lysosomal compartment leads to red fluorescence, which dissipates when the dye leaks from this compartment into the cytosol^43^. Indeed, treatment of fibroblasts with air plasma and ozone did not induce a significant decrease in the AO fluorescence (Fig. 7a, b) indicating the absence of lysosomal permeabilization. Additionally, helium plasma had no significant effect on lysosomal permeabilization (Fig. 7a, b). However, lysosomal acidification was revealed by the increasing detectability of AO fluorescence 4 h after helium plasma exposure (Fig. 7b).

The depolarization of the mitochondrial membrane and concomitant change of the mitochondrial potential (ΔΨ_m_) has been implicated as an early event in the process of cell death^30^,^44^. The induction of mitochondrial membrane surface charge dissipation and corresponding change of ΔΨ_m_ is sufficient to trigger apoptosis or necrosis^30^,^44^. Thus we used the fluorescent dye JC-1 to investigate whether air, helium plasma or ozone may perturb mitochondrial function. JC-1 represents a cationic dye that exhibits a potential-dependent accumulation in mitochondria, which can be monitored by a shift in fluorescence emission from green to red using fluorometer^45^. As expected, both plasmas and ozone induced depolarization of the mitochondrial membrane as indicated by a decrease of the red-to-green fluorescence intensity ratio as revealed by fluorometric measurements (Fig. 7c) and confirmed using flow cytometry (see Fig. S2 in the Supplementary Information).

### The Stopping and Range of Ions in Matter (SRIM); simulations of ions penetration through the cell membrane

The discrepancy over subcellular effects between helium and air plasma, inspired us to search for physical reasons for such differences. To understand the physical and biological (biochemical) reasons for plasma-induced cell death and grasp the difference in mechanisms of helium and air plasmas actions, we analyzed ion penetration profiles in the model system, consisting of the three laterally infinite layers: water, cell membrane and water. The number of ions per unit of plasma volume was estimated as *n* = 5·10^17^ m^−3^ (see the Methods section). During the treatment time Δt = 15, sec the plasma with an ion density *n* = 5·10^17^ m^−3^ and gas flow rate 4 liter min^−1^ produces the mean ion fluence, *F_15_* = 1.6·10^14^ ions cm^−2^. To obtain fluence for 30 sec and 60 sec treatments, the fluence *F_15_* should be multiplied by a factor of 2 or 4, accordingly. The SRIM simulations shown in Figure 7d indicate that the ion distributions exhibited a nearly Gaussian lateral profile with the mean ion ranges and widths at half amplitude: for helium (He) ions 11.6 nm and 5.8 nm, and for nitrogen (N) ions 4.8 nm and 2.1 nm, respectively. Moreover, during the irradiation a significant amount of He ions accumulated at the water/membrane interface, while it is still unreachable for N ions. Qualitatively, the same picture would be expected for any heavy ions of air plasma as they have a shorter penetration range than He ions (Fig. 7d). Thus, the simulated in-depth profiles of the plasma ion distributions in a medium-cell system give an indication as to the differences in cell surface morphology (Fig. 5). Specifically, irradiation of cells by lighter He ions leads to a longer penetration profile than that of heavier N or O ion irradiation, resulting in observed differences in membrane roughness between helium and air plasma. Moreover, differences in ion penetration profiles could be the underlying physical reason behind biochemical outcomes of different plasma types.

### Scavenging intracellular ROS abolishes the cytotoxic effects of air, helium plasma and ozone

To confirm the role of ROS in the induction of cell death and subsequent events by both air and helium plasmas as well as ozone, we used the ROS-scavenger *N-acetyl-L-cysteine*. *N-acetyl-L-cysteine*, a derivative of the dietary amino acid *L-cysteine*, is known to be a powerful free radical scavenger and has profound cytoprotective effects against ROS-induced cell death^46^,^47^. Furthermore, our previous study demonstrated the applicability of *N-acetyl-L-cysteine* as a free radical scavenger^48^. As expected, by scavenging intracellular ROS/RNS we were able to antagonize the cytotoxic effects elicited by both plasmas and ozone on fibroblasts. As shown in Figure 7e, treatment with *N-acetyl-L-cysteine* decreased the cytotoxicity of air and helium plasmas and ozone leading to completely abolished cytotoxic effects 24 h after exposure.

## Discussion

Nowadays it is becoming clear that non-thermal plasmas have undoubtedly great potential. However, clinical applications of non-thermal plasmas are restrained due to the lack of knowledge about the molecular mechanisms of plasma-living cell interactions. Moreover, there is no information comparing the consequences of different plasma treatments on cells with pure ozone treatment (an abundant component of non-thermal plasmas). Thus, we selected two distinct plasmas (air and helium) for our study.

Our comparative study showed air plasma and ozone to have very high bactericidal potency (Fig. 2a,b), both producing nearly 99.99% efficacy. Moreover, both air plasma and ozone inactivated different bacterial strains *P. aeruginosa* and *S. aureus* to the same extent (Fig. 2a, b). In contrast, bacteria treatment using helium plasma had a much lower effectivity, reaching at most only 8.71% of bactericidal efficacy. Furthermore, helium plasma showed higher efficacy in killing Gram-negative *P. aeruginosa* rather than Gram-positive *S. aureus* bacteria (Fig. 2c,d). Interestingly, the cell wall of Gram-negative bacteria is composed of a thin layer of peptidoglycans, which itself is surrounded by an outer membrane containing lipopolysaccharide. In contrast, the Gram-positive bacteria envelop does not contain an outer membrane but is surrounded by layers of peptidoglycan many times thicker than in Gram-negatives^49^,^50^. On the other hand, from a physical point of view, under plasma ion bombardment the penetration depth of light ions (like He) is larger than that of heavier ions (ions of air). Thus, the differences in bacterial cell wall thickness do not, however, explain the similar effects of air plasma in *P. aeruginosa* and *S. aureus* deactivation.

We suppose that different cell wall chemical resistance and/or respiration mechanisms may play a key role in the sensitivities of these bacteria to the plasmas. Tentative explanations for the different efficacies of *P. aeruginosa* and *S. aureus* deactivation by He and air plasmas are as follows: 1) membrane damage by ion and electron bombardment; 2) membrane perforation by etching due to highly reactive species; 3) electrostatic disruption of cell parts due to mutual electrostatic repulsion of the retained ions.

In this study we show that both, air and helium plasma as well as ozone produce intracellular ROS and RNS, accumulation of which leads to cell death. Our study is in line with previous studies^14^,^15^,^16^ identifying the potential role of ROS and RNS in the underlying biological effects of plasma. Interestingly, ozone showed higher cytotoxicity in comparison to air and helium plasma, having drastic toxicity even immediately after exposure (Fig. 3a). This indicates that ozone possesses unspecific cellular responses. In line with toxicity data, ozone rapidly triggered ROS/RNS formation more efficiently than both plasmas (Fig. 4a, b). However, later helium plasma was more potent than ozone and air plasma in the generation of superoxide (Fig. 4d). Indeed, ROS and RNS are key effectors in signal transduction^51^. When ROS is produced during normal physiological processes, they are rapidly scavenged by antioxidant enzymes. Hence, excess of ROS can induce apoptotic/necrotic cell death through prolonged activation of JNK signalling^39^,^51^. Furthermore, ROS/RNS may lead to an increase in the permeability of the outer mitochondrial membrane and damage of lysosomal membranes^52^.

Interestingly, the glioma cell line developed much lower toxic effects in response to ozone and both plasmas in comparison with fibroblasts (Fig. 3b). This observation can be explained by the fact that cancer cells have an upregulated antioxidant capacity developed in response to intrinsic oxidative stress^53^. This leads to the resistance of cancer cells to ROS-dependant drugs^53^. Suppression of ROS elimination mechanisms in combination with the application of ROS-generating agents (such as non-thermal plasma) could result in a potent strategy to enhance cancer cell cytotoxicity.

We have demonstrated that exposure to plasmas led to delayed alteration in membrane morphology, while ozone treatment induced immediate dramatic changes in the cell surface (Fig. 5a). Following the treatment of cells with both plasmas, the outer cellular membrane increased in roughness (Fig. 5b, c), resulting in disruption of membrane integrity. Similarly, an increase in roughness of HeLa cellular membranes 3–6 h post air plasma jet treatment was recently revealed by AFM^54^.

Numerous studies have shown that lysosomal rupture and subsequent lysosomal enzymes leakage may lead to the production of oxidants in the mitochondria^41^,^42^. In fact, air and helium plasma as well as ozone treatment of fibroblasts resulted in the depolarization of the mitochondrial membrane (Fig. 6c). However, our results demonstrate that upon air or ozone application there is no significant effect on lysosomal membrane permeability (Fig. 6a,b). Interestingly, helium plasma exposure resulted in considerable lysosomal acidification (Fig. 6b). Recently it has been found that suppression of the mammalian target of rapamycin (mTOR) activity leads to enhanced acidification of lysosomes and subsequent autophagy induction^55^. Simulations of ion penetration through the cell membrane revealed that heavy ions of atmospheric plasma have a shorter penetration range than He ions. Such ion distribution and related ionization loss may enhance the temperature of the first and second layers, lead to the acceleration of diffusion processes and creation of ROS. Generally, our results imply that notwithstanding of having similar cytotoxicity by air and helium plasma (Fig. 3a,b) there is discrepancy in the superoxide accumulation, lysosomal activity and plasma membrane roughness. These findings suggest that triggered signalling cascades are different upon air and helium plasma treatment of living cells. Furthermore, ozone treatment showed that this compound is extremely reactive, showing significant toxicity with short term exposure of fibroblasts (Fig. 3a). In contrast, both plasmas had delayed toxic effects on fibroblasts. Nonetheless, despite inducing acute cellular damage the antimicrobial effects of ozone were of the same order as air plasma (Fig. 2a,b). This finding indicates that the potential application of air plasma in infectious wound healing might be advantageous over ozone treatment as it possesses significantly lower side effects on living tissue.

Furthermore, we showed that ROS scavenger *N-acetyl-L-cysteine* reduced cytotoxicity caused by ROS production induced by air, helium plasma and ozone indicating that dietary supplementation with antioxidants might be a suitable strategy to reduce oxidative damage. These data reveal the crucial role of ROS/RNS in triggering intracellular signalling by plasma.

In summary, our study shows that the antibacterial efficacy of air plasma was comparable to ozone, whereas helium plasma possessed significantly lower antibacterial effects. Furthermore, ozone exhibits dramatically higher toxic effects on living cells than air or helium plasma. Indeed, the extent of cytotoxicity may grossly differ between fibroblasts and phenotypically distinct tumour cell lines. Moreover, despite having the same cytotoxicity upon air and helium plasma treatment, our findings imply that the triggered signalling cascades may be grossly different. This indicates that these processes depend critically on the chemical composition of the plasma jet. In addition, proteins of the mTOR signalling pathway, which are overexpressed or deregulated in human cancers, represent a target for cancer therapy^56^, and a link between the deregulation of lysosomal activity and mTOR inhibition has been revealed^55^,^57^. In light of these facts our data on lysosomal acidification by helium plasma are of great value and bring new perspectives for the development of novel therapeutic strategies of cancer.

These results imply that the cytotoxic effects of non-thermal plasma require more intensive study in terms of the identification of molecular targets of two distinct types of plasmas and that they should be considered in biomedical applications.

## Methods

### Physicochemical characterization of the plasma

To produce uniform non-thermal plasma for biological applications, we utilized the plasma setup shown in Figure 1a. The input voltage was about 600 V, electric current 167 mA and the power was 100 W; such a high voltage supply resulted in electron energy of about 0.5 keV. The schematic construction of the plasma jet nozzle is shown in Figure 1e, consisting of a plasma generation module and cases. The high voltage electrode, a porous ceramic membrane and ground electrode form a plasma generation module. The gas supply was administered through a gas inlet followed by gas ionization in the pores of the ceramic membrane utilizing an electric field between two electrodes. The gas temperature at the tip of the plasma jet was measured using a thermocouple embedded in an optical spectrograph USB 4000 (Ocean Optics Inc.). The temperature remained 37–40°C during the cell treatment. The optical emission spectrum of the non-thermal plasma was measured using an optical spectrograph USB 4000 (Ocean Optics Inc.).

### Cell culture

3T3 fibroblasts and a C6 glioma cell line (American Type Culture Collection) were grown in culture medium containing Dulbecco's modified Eagle's medium (DMEM; PAA Laboratories, Pasching, Austria) with 10% fetal bovine serum (FBS; PAA Laboratories) and Primocin TM (100 µg/ml; Lonza, Cologne, Germany). Cells were cultured in a humidified 5% CO_2_ atmosphere at 37°C.

### Plasma treatment

Non-thermal plasma was produced using an experimental custom-designed system with a nozzle array to treat living cells as illustrated by Figure 1e. In order to alter the chemical composition of the plasma, we used either an air or helium gas supply. Gas was supplied through a gas inlet and then was ionized in the pores of the ceramic membrane. To compare effects of plasma with ozone, cells were exposed to ozone produced by an ozone generator with input voltage 230 V, the power 12 W and ozone gas production rate 400 mg h^−1^. Cells grown to 70% confluence were exposed to plasma from the device located 10 mm away, for 15, 30 and 60 s. Before the treatment, the medium was removed from culture wells and then 50 μl of medium was added to prevent cells from drying up. After the plasma treatment, the remaining medium was replaced with the fresh one.

### Cultivation of bacteria and inactivation by plasma

To study bactericidal effects of plasma we used the Gram-negative *Pseudomonas aeruginosa* (ATCC 27853) and Gram-positive *Staphylococcus aureus* (ATCC 6538) bacterial strains (Czech Collection of Microorganisms (Brno, Czech Republic). The gelatine pellets containing the bacterial strains were incubated in 9 ml of liquid media (Tryptic Soy Broth, Mecrotube®, Merc, NJ, USA) at 35°C for 18 hours, and then diluted in a phosphate buffer (PBS) to a concentration 6 × 10^6^ colony forming units (CFU)/ml. A volume of 0.8 ml of diluted bacteria suspension was spread onto the agar plate (Caso-Agar, Mercoplate®, Merc) and exposed to plasma or ozone from the device located 10 mm away, for 15, 30 60 s. The plates were incubated overnight and the number of CFU in the 16 cm^2^ inhibition zone was counted using ImageJ software (National Institutes of Health, Bethesda, MD, USA). The tested layers were tested in triplicates. Bactericidal efficacy was calculated according to the following formula: 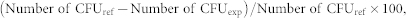
where CFU_ref_ and CFU_exp_ are the number of living bacteria colonies in the untreated and treated group, respectively.

### Measurement of cellular viability

Cell viability was analyzed by WST-1 assay (Roche Diagnostics), which is based on the cleavage of tetrazolium salt WST-1 by cellular mitochondrial dehydrogenases, producing a soluble formazan salt; this conversion only occurs in viable cells, thus allowing accurate the spectrophotometric quantification of the number of metabolically active cells in the culture. In order to block ROS/RNS, the ROS scavenging agent 5 mM *N-acetyl-L-cysteine* (NAC) was added to the complete culture medium. 3T3 fibroblasts were seeded onto 96-well plates at a density of 8000 cells per well and treated with plasma or ozone. Immediately or 24 h after the treatment, WST-1 reagent was added to each dish and incubated for 2 h at 37°C to form formazan. The absorbance was measured using a Tecan-Spectra ELISA plate reader (Mannedorf, Switzerland) at 450 nm. Readings were done in quadruplicates; three independent experiments were performed for each measurement.

### Detection of intracellular ROS and RNS

ROS and RNS levels were measured using Cellular ROS/Superoxide Detection Assay Kit (Abcam). Briefly, cells were seeded onto 96-well black/clear bottom plates (Corning, BD Biosciences) at a density of 8000 cells per well. Following this, plasma treatment cells were labeled with Oxidative Stress Detection Reagent (Green) for ROS detection and Superoxide Detection Reagent (Orange) according to the manufacturer's instruction (Abcam). Indeed, the green probe reacts directly with a wide range of reactive species, such as hydrogen peroxide (H_2_O_2_), peroxynitrite (ONOO^−^), hydroxyl radicals (HO), nitric oxide (NO), and peroxyradical (ROO) that allowed us to measure the total level of different ROS/RNS types. In contrast, the orange probe reacts specifically with superoxide (O_2_^−^), allowing for the detection of elevated superoxide levels. Fluorescence was then measured using a fluorescent microplate reader (Tecan Infinite® 200 PRO). Readings were done in quadruplicates. Quantification of ROS levels was done using methods published earlier^58^.

### Apoptosis assay

Apoptosis was assessed via annexin V/propidium iodide staining. Cells were treated with different plasmas and ozone for 15, 30 and 60 s as indicated and incubated further for 4 h. Phosphatidylserine expression as an early sign of apoptosis was determined via fluorescence microscopy analysis buy the binding of fluorescein isothiocyanate-labeled annexin V (Sigma-Aldrich); propidium iodide was used to differentiate necrotic cells. Fluorescence images were recorded with a Zeiss Axioscope 2 microscope (Carl Zeiss AG). ImageJ software was used for image processing and fluorescent micrograph quantification.

### Quantification of mitochondrial membrane potential

Plasma-treated cells were further incubated for 4 h to measure mitochondrial membrane potential (ΔΨ_m_). After 4 h of incubation, cells were loaded with 1 µM JC-1 (Invitrogen), a lipophilic cationic fluorescence dye with a dual emission wavelength for 30 min, in order to analyze the depolarization of the ΔΨ_m_. At low concentrations (due to low ΔΨ_m_) JC-1 is predominantly a monomer that results in a green fluorescence with emission of 530 nm. At high concentrations (due to high ΔΨ_m_) the dye aggregates, yielding a red emission of 590 nm. Thus a decrease in the aggregate fluorescent count displays a mitochondrial membrane depolarization whereas an increase exhibits a hyperpolarization. Following staining, cells were analyzed using a fluorescent microplate reader (Tecan Infinite® 200 PRO). Readings were done in quadruplicates. Additionally, flow cytometry was performed using a Apogee Flow Cytometer (Auto 40).

### Assessment of lysosomal integrity by acridine orange (AO) release

Cells were labeled with 5 µg/ml AO in DMEM culture medium for 15 min at 37°C. After the rinsing of cells in complete medium, they were exposed to different types of plasma and ozone. Following this, cells were cultured at 37°C for 4 h and then, orange fluorescence intensity was measured using a fluorescent microplate reader (Tecan Infinite® 200 PRO). Readings were done in quadruplicates. Fluorescence images were recorded with a Zeiss microscope Axioscope 2 (Carl Zeiss AG) and ImageJ software (NIH) was used for image processing.

### Stopping and Range of Ions in Matter (SRIM) simulations

Stopping and Range of Ions in Matter (SRIM) simulations^59^ are the most common way of obtaining a rough view of the in-depth composition and the amount of implanted ions in the layered structures. Simulations were performed for helium and atmospheric plasmas with an ion energy of 0.5 keV and typical fluences on the three-layers H_2_O (10 nm)/membrane (8 nm)/H_2_O (10 nm). In the helium plasma, electron and ion concentration can be calculated as^60^

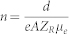
where *d* is the length of the resistive plasma jet column, *e* is the charge of the electron, *A* is the cross section of the plasma jet, *Z_R_* is the real part of the single plasma jet column impedance and µ_e_ = 1.13·10^−1^ m^2^ V^−1^ s^−1^ is the electron mobility in helium at atmospheric pressure (10^5^ Pa). An estimation was made using the described equation for the measured impedance *Z_R_* = 3600 Om, *d* = 10 mm, and *A* = 10 mm^2^ gives the ion concentration, n = 5·10^17^ m^−3^.

### Atomic Force Microscopy (AFM)

The effect of cell exposure to air and helium plasma and ozone on changes in cell membrane morphology was studied by AFM. A glass slide with fixed cells was removed from PBS, rinsed with MilliQ-water several times and dried in the laminar flow-box for 30 min. All images were acquired with Atomic Force Microscope Dimension Icon (Bruker) as topographical scans in Peak Force tapping mode under ambient conditions, using etched silicon tips MPP (TAP150A, Bruker). The images were taken with a scan rate within the range of 0.25–0.9 Hz. The images were analyzed using NanoScope Analysis software (Bruker). The 10 × 10 µm scans were fitted to the plane or flattened in order to remove the tilt of rough data and then the roughness of the membrane was determined. Five cells of each treatment type have been analyzed by randomly measuring several areas over each cell surface.

### Statistical analysis

Quantitative results are expressed as mean ± SEM. Results were analyzed by multi-group comparison Fisher's LSD and Newman-Keuls tests. Differences were considered statistically significant at *P < 0.05.

## Author Contributions

O.L., V.Z., S.K. and A.D. designed experiments and interpreted data; O.L. and S.K. carried out most of experiments; E.C. performed AFM imaging and analysis; E.S. provided critical input to the overall research direction; O.C. designed the low temperature plasma system; O.L., V.Z. and S.K. wrote the manuscript with input from all co-authors.

## Supplementary Material

Supplementary Information Supplementary Information
