# Supplementary material for: Cell death induced by ozone and various non-thermal plasmas: therapeutic perspectives and limitations
Source: Sci Rep. 2014 Nov 20;4:7129. doi: 10.1038/srep07129 (PMC4238021; doi:10.1038/srep07129)
Supplement: Supplementary Information [file srep07129-s1.pdf]

Supplementary Information

Cell death induced by ozone and various non-thermal plasmas: therapeutic perspectives and limitations

Oleg Lunov[1], Vitalii Zablotskii[1], Olexander Churpita[1], Eliška Chánová[2], Eva Syková[3], Alexandr Dejneka[1] and Šárka Kubinová[1,3]

[1]Institute of Physics AS CR, Prague, Czech Republic

[2]Institute of Macromolecular Chemistry AS CR, Prague, Czech Republic

[3]Institute of Experimental Medicine AS CR, Prague, Czech Republic

Correspondence and requests for materials should be addressed to O.L. (email: lunov@fzu.cz).

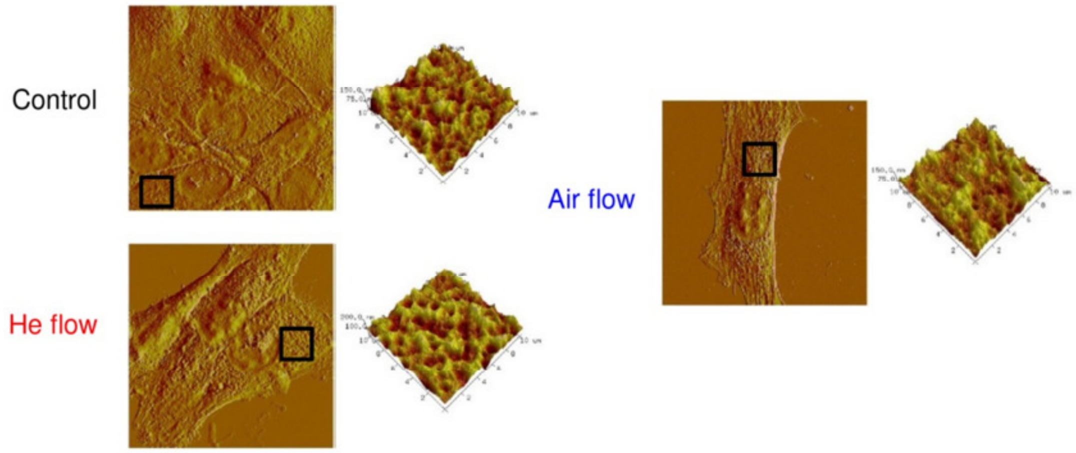

Supplementary Figure S1 AFM analysis of cell membrane integrity. 3T3 fibroblasts were treated with air, helium flow (no plasma treatment), and then 4 h after treatment cells were fixed and imaged with AFM microscopy. Images represent a 90 x 90 µm area of scan. The black square areas indicate where a 3D 10 x 10 µm scan has been performed.

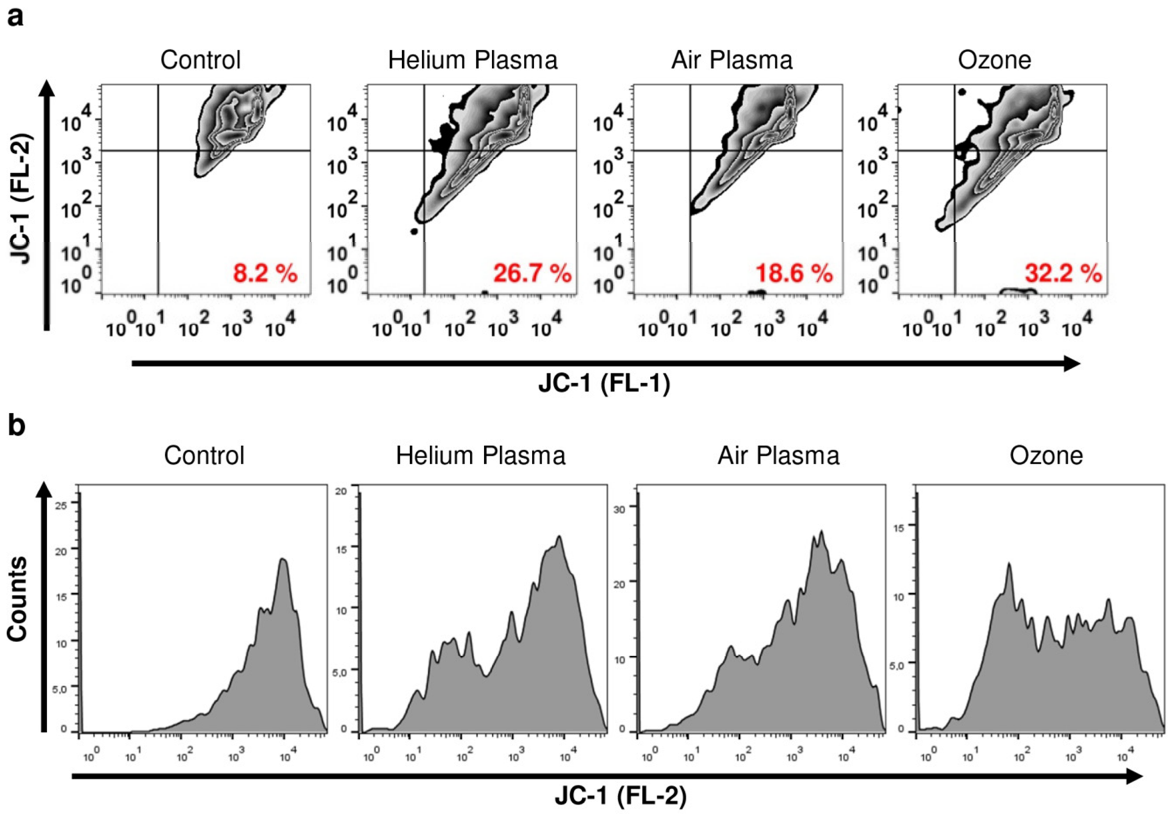

Supplementary Figure S2 Air, helium plasma and ozone effects on mitochondrial metabolic activity of 3T3 fibroblasts 4 h after treatment. (**a**, **b**) Assessment of mitochondrial membrane integrity state in treated cells with air and helium plasma in comparison with ozone. Cells were stained with JC-1 probe, the JC-1 fluorescence was measured using flow cytometry.
